# Identifying Triggers of Alcohol Craving to Develop Effective Virtual Environments for Cue Exposure Therapy

**DOI:** 10.3389/fpsyg.2019.00074

**Published:** 2019-01-29

**Authors:** Alexandra Ghiţă, Lidia Teixidor, Miquel Monras, Lluisa Ortega, Silvia Mondon, Antoni Gual, Sofia Miranda Paredes, Laura Villares Urgell, Bruno Porras-García, Marta Ferrer-García, José Gutiérrez-Maldonado

**Affiliations:** ^1^Department of Clinical Psychology and Psychobiology, University of Barcelona, Barcelona, Spain; ^2^Addictive Behaviors Unit, Hospital Clinic of Barcelona, Barcelona, Spain

**Keywords:** alcohol use disorder, alcohol craving, virtual reality, cue exposure therapy, ALCO-VR

## Abstract

**Background:** Many studies have indicated that alcohol craving is a core mechanism in the acquisition, maintenance, and precipitation of relapse in alcohol use disorder (AUD). A common treatment approach in AUD is cue exposure therapy (CET). New technologies like virtual reality (VR) have the potential to enhance the effectiveness of CET by creating realistic scenarios in naturalistic environments. In this study, we aimed to determine relevant triggers of alcohol craving in patients with AUD.

**Methods:** We enrolled 75 outpatients diagnosed with AUD according to the DSM-5 criteria Participants completed the Alcohol Use Disorder Identification Test and a self-administered questionnaire to assess alcohol craving. The variables included in the craving questionnaire were as follows: presence of others, situations, time of the day, day of the week, mood, and type of alcoholic beverage.

**Results:** Greater levels of alcohol craving were seen in many situations, including being at a party, in a restaurant, in a bar or pub, and at home. Drinking alone and drinking with two or more friends were equally associated with higher levels of craving. Drinking at night and drinking at weekends also emerged as triggers for alcohol craving. Emotional states like anxiety or tension, sadness, stress, frustration, or irritability were highly associated with urges to drink alcohol. The alcoholic drinks most highly associated with increased levels of craving were beer, wine, and whisky. Gender and age implications were discussed.

**Conclusion:** This study is part of a larger project aiming to develop and validate CET based on VR technology for patients with AUD who are resistant to classical treatment. The identified triggers have been used to develop relevant VR environments for CET, and further research is ongoing to implement our findings.

## Introduction

There is extensive research suggesting that alcohol craving is a core mechanism in the acquisition, maintenance and precipitation of relapse in alcohol use disorder (AUD) (Bordnick et al., 2008; Papachristou et al., 2012; Sinha, 2013; Schacht et al., 2013; Kim and Lee, 2015; van Lier et al., 2018). It is also an important criterion in the diagnosis of AUD (American Psychiatric Association [APA], 2013). Craving alcohol reflects a pathological state (Huang et al., 2018), an intense urge to consume alcohol (Serre et al., 2015), that is sustained by impaired inhibitory control to abstain (Stein et al., 2018). According to a recent systematic review by van Lier et al. (2018), alcohol craving is a multidimensional construct related to specific cognitive processes, including attentional bias toward alcohol-related content (Manchery et al., 2017), implicit beliefs regarding alcohol consumption (Monk and Heim, 2013), conditioning processes (van Lier et al., 2018), psychobiological arousal and responses (Schacht et al., 2013), and motivational approach tendencies (Noyes and Schlauch, 2018). These models have provided the framework for current research in alcohol craving (van Lier et al., 2018).

The *situational specificity* hypothesis (Wall et al., 2001) explains that alcohol craving is determined by naturalistic drinking cues and environments (Monk and Heim, 2013). This contextual dependency in alcohol craving is reflected by the cue-reactivity paradigm (Coffey et al., 2006; Hone-Blanchet et al., 2014; Ramirez et al., 2015a; Qureshi et al., 2017; Stein et al., 2018), where alcohol-related stimuli emerge as risk factors for binge drinking episodes (Ryan et al., 2010) and AUD (Stein et al., 2018), with the potential to facilitate relapse even after a prolonged period of abstinence (Weerts et al., 2006; Robinson and Berridge, 2008). Hence, identifying alcohol-related cues and contexts is fundamental to cognitive and behavioral intervention, as well as addressing real-life factors that impede long-term abstinence (Pericot-Valverde et al., 2015). The cue-reactivity paradigm led to the development of the cue exposure therapy (CET) (Ramirez et al., 2015b).

Cue exposure therapy is a therapeutic approach that uses repeated exposure to stimuli that provoke craving (Loeber et al., 2006), aiming to prevent future relapses. The rationale for CET is based on the Pavlovian conditioning model (Pavlov, 1927). In alcohol misuse, conditioned processes are established between alcohol as stimuli and neutral cues over prolonged and repeated pairings (Craske et al., 2014), accompanied by positive reinforcement (Vollstädt-Klein et al., 2011). Similarly, exposure to conditioned stimuli elicits psychophysiological responses experienced as increased alcohol craving (Bordnick et al., 2011). The goal of CET is to extinguish alcohol craving by suppressing the link between the conditioned stimuli, in this case alcohol, and the unconditioned stimuli (Watson et al., 2011), by building coping skills (Giovancarli et al., 2016) that increase self-efficacy (Barlow et al., 2016). However, a meta-analysis by Conklin and Tiffany (2002) concluded that CET results were inconsistent, and they explained that most treatments were carried out by presenting only one cue at a time in a controlled setting. This meant that it was difficult to generalize the effects of such therapy to real-life situations. New technologies like virtual reality (VR) have the potential to enhance the effectiveness of CET by creating realistic scenarios that more closely resemble natural environments (Wiederhold and Rizzo, 2005).

Virtual reality technology simulates real-life settings by applying multiple sensory inputs (i.e., visual, auditory, olfactory, or tactile), disorder-related cues, and scenarios to create fully immersive experiences (Riva, 2009). The resulting environments promote social interaction, particularly related to substance use, engaging the individual’s sense of presence within the environments (Riva et al., 2007). Such human-computer interaction enhances ecological validity by allowing the generalization of newly learned skills to naturalistic environments (Rizzo et al., 2004), ultimately benefitting traditional methods like CET (Schultheis and Rizzo, 2001). As a current focus in psychological research, VR technology is increasingly being applied as an assessment or treatment instrument in anxiety disorders (Krijn et al., 2004; Powers and Emmelkamp, 2008; Meyerbröker and Emmelkamp, 2010; Opriş et al., 2012; Maples-Keller et al., 2017), psychosis (Freeman, 2008; Rus-Calafell et al., 2017), and eating disorders (Ferrer-García and Gutiérrez Maldonado, 2012; Marco et al., 2013; Mölbert et al., 2017), as well as for cognitive or social training in autism spectrum disorder (Kandalaft et al., 2013; Ip et al., 2018), dementia (Blackman et al., 2007), and depression (Dehn et al., 2017).

In the field of substance use disorders, VR has been used to explore drug craving in opioid use disorder (Kuntze et al., 2001), tobacco use disorder (Bordnick et al., 2012; Pericot-Valverde et al., 2014), and stimulant use disorders, including cocaine (Saladin et al., 2006) and methamphetamine (Culbertson et al., 2010). In AUD, six studies have used VR as an assessment tool to explore alcohol cravings (Bordnick et al., 2008; Cho et al., 2008; Lee et al., 2008; Ryan et al., 2010; Choi and Lee, 2015; Kim and Lee, 2015) and five studies have examined the effectiveness of VR exposure therapy (VRET) for reducing the urge to consume alcohol (Kwon et al., 2006; Lee et al., 2007, 2009; Hyun et al., 2013; Son et al., 2015). The results of these studies are consistent and indicate that VRET shows promise for the treatment of AUD (Ghiţă and Gutiérrez-Maldonado, 2018).

Many factors contribute to alcohol craving, including alcohol-related stimuli (e.g., the sound of glasses in a bar) (Lee et al., 2008) or emotional factors (e.g., a determined mood that precipitates alcohol consumption) (Wardell and Read, 2013). It has been suggested that alcohol craving is also triggered by peer pressure in social environments (Litten et al., 2015). Nevertheless, each individual, depending on his or her previous experience of alcohol consumption, develops a particular association with pre-determined triggers of alcohol craving (Sinha et al., 2009). It has consistently been reported that alcohol cravings are context-dependent (Drummond, 2001; Zironi et al., 2006; Steketee and Kalivas, 2011; Monk and Heim, 2013; Qureshi et al., 2017). Given that alcohol stimuli are prevalent in society, especially in Western cultures, there is a much higher likelihood of patients with AUD experiencing relapse (Zironi et al., 2006; Witteman et al., 2015), even after several treatments (Streeton and Whelan, 2001; Charney et al., 2010). Clinicians and scientists are therefore changing their emphasis to developing treatment approaches that are focused on alcohol-based naturalistic contexts using modern technologies like VR or augmented reality. Among the factors that need to be considered in these approaches are the roles of alcohol-related contexts and cues, social interaction, emotional valence, and the type of alcoholic beverage, among other lesser triggers of alcohol craving. These warrant further discussion for the current research.

Alcohol cravings increase when individuals are exposed to alcohol-related contexts. The most common alcohol-related contexts are pubs (Townshend and Duka, 2001; Wall et al., 2001; Loeber et al., 2006; Lee et al., 2007, 2008, 2009; Cho et al., 2008; Gatti et al., 2008; Nees et al., 2012; Monk and Heim, 2013; Kim and Lee, 2015; Parsons, 2015; Christiansen et al., 2017; Qureshi et al., 2017), restaurants (Kwon et al., 2006; Cho et al., 2008; Gatti et al., 2008; Lee et al., 2009; Hone-Blanchet et al., 2014; Spagnoli et al., 2014; Parsons, 2015; Son et al., 2015; Qureshi et al., 2017), bars (Lee et al., 2007; Bordnick et al., 2008; Chaplin et al., 2008; Sinha et al., 2009; Miller and Fillmore, 2010; Monk and Heim, 2013; Hone-Blanchet et al., 2014; Christiansen et al., 2017), kitchens or other rooms in the house (Kwon et al., 2006; Bordnick et al., 2008; Ryan et al., 2010; Garland et al., 2012; Hone-Blanchet et al., 2014; Spagnoli et al., 2014), parties (Oei and Morawska, 2004; Bordnick et al., 2008; Miller and Fillmore, 2010; Ryan et al., 2010; Wardell and Read, 2013), within the house (Bordnick et al., 2008; Nyaronga et al., 2009), parks and other public spaces (Nyaronga et al., 2009), and during meals (Voller and Allamani, 2014).

Alcohol-related behaviors are highly associated with social environments, especially in college students, but also in light drinkers, heavy drinkers (Ryan et al., 2010), and patients diagnosed with AUD. In a systematic review by Monk and Heim (2013), drinking with peers was reported to increase the urge to consume alcohol and that individuals tended to have greater alcohol intakes when accompanied than when alone (Cho et al., 2008). Social pressure is an important factor in alcohol craving, and Lee et al. (2008) suggested that it may have a greater triggering potential than alcohol cues itself in binge drinkers or heavy drinkers, but that this pattern was not maintained in patients with AUD, where both social pressure and exposure to alcohol-related stimuli were associated with increased levels of alcohol craving. Similarly, cravings and loss of control may result into varied drinking patterns among patients with AUD, including drinking alone (Monk and Heim, 2013). Nevertheless, the simple presence of peers may promote alcohol craving (Drummond, 2001) and precipitate consumption in patients with AUD. Monk and Heim (2014) proposed exploring whether individuals experienced increased alcohol craving when alone and when accompanied by one or more friends, relatives, work mates, or their partners.

Mood and the emotional valence of a given situation may elicit alcohol craving and intake (Wardell and Read, 2013). In particular, patients with AUD are at great risk for relapse because they have increased vulnerability and fewer coping skills in stressful environments (Sinha, 2008). In other addictive behaviors, Pla-Sanjuanelo et al. (2015) proposed that certain moods should be treated as triggering factors for craving, focusing on boredom, stress, anxiety or tension, irritability, frustration, sadness, anger, depression, calmness, happiness, and euphoria. Similarly, Wiers et al. (2003) suggested that subjective cognitive states such as relaxation, pride, embarrassment, fatigue, disgust, or distress were highly associated with alcohol misuse patterns.

According to the Spanish National Survey on Alcohol Consumption in 2012 (Encuesta Nacional de Salud, 2011/2012), beer was the preferred alcoholic drink in the general population, followed by wine and liquors. Howland et al. (2008) mentioned alcoholic drinks like rum, vodka, gin, bourbon, and other liquors, as well as beer and different wines. In a study by Choi and Lee (2015), “soju” was mentioned, which is a Korean alcoholic beverage. However, the present study is more related to Western cultures, and so refers to culture-related alcoholic beverages.

Finally, other factors contributing to alcohol craving include alcohol-related sounds, sights, and smells (Wiers et al., 2003; Qureshi et al., 2017). Other important variables that may modulate alcohol craving are the time of the day (van Lier et al., 2018) and the day of the week (Craske et al., 2014).

In a previous project at the VR-Psy Lab at the University of Barcelona, we aimed to develop a VR-based treatment for patients diagnosed with bulimia nervosa (BN). First, we performed exhaustive interviews with patients with BN to explore and identify the triggers for binge eating behaviors (Pla-Sanjuanelo et al., 2015). Based on those results, VR environments were created and validated in patients with BN (Pla-Sanjuanelo et al., 2017) as a prelude to developing a VR-cue exposure treatment. Finally, a clinical trial was conducted to explore the efficacy of the VR-based treatment versus cognitive-behavioral therapy (CBT) in patients with BN (Ferrer-García et al., 2017). At post-treatment assessment, there were significant differences between groups in terms of abstinence rates, anxiety and food craving, which highlights the efficacy of VR-CET over CBT.

The current study is part of a larger project aiming to develop and validate VR alcohol-related environments as a treatment instrument for patients diagnosed with AUD. A common procedure for developing ecologically valid VR environments is to conduct exhaustive interviews with study-relevant individuals (Reger et al., 2009); an example is Kwon et al. (2006). Based on the literature, we developed a questionnaire to assess levels of craving associated with different variables that may trigger alcohol craving in patients diagnosed with AUD. We were particularly interested in the interplay between different self-reported ratings of craving related to preferred alcoholic drinks and contexts. In addition, we focused on assessing gender differences in terms of self-reported craving levels. Finally, we also explored age differences in all the variables included in the questionnaire.

## Materials and Methods

### Participants

The sample consisted of 75 outpatients [52 males, 23 females; mean age 53.9 years, standard deviation (*SD*) = 11.27, range 22–74], all of whom met the criteria for AUD set out in the *Diagnostic and Statistical Manual of Mental Disorders* (5th ed., American Psychiatric Association [APA], 2013) and provided their written informed consent to participate. The ethics committees of the University of Barcelona and the Hospital Clinic of Barcelona (Spain) approved this study. Additional inclusion criteria were that they were to be older than 18 years and to be receiving ambulatory treatment at the Addictive Behaviors Unit of the Hospital Clinic of Barcelona. Patients with comorbid psychotic disorders and/or patients with severe cognitive deficits were excluded.

### Instruments

#### Alcohol Use Disorder Identification Test (Saunders et al., 1993)

The Spanish version of the AUDIT (Contel Guillamón et al., 1999) is a screening instrument that explores harmful alcohol use. It consists of a 10-item questionnaire to which responses are scored on a scale from 0 to 4, giving a maximum score of 40. This inventory showed good internal consistency, with a Cronbach’s α coefficient of 0.88. The AUDIT is one of the most utilized instruments for detecting the severity of drinking behavior patterns.

#### The Self-Administered Craving Questionnaire

Because multiple variables are associated with the desire to drink alcohol, we developed a questionnaire to assess craving levels. We included the following variables: *presence of others* (i.e., drinking alone, drinking with a friend, drinking with two or more friends, drinking with a partner, drinking with relatives, and drinking with co-workers), *situations* (i.e., kitchen, living room, work place, restaurant, bar, pub, supermarket, park, club, party, at home, during meals, or bedroom), *time of the day* (i.e., morning, midday, afternoon, or night), *day of the week* (i.e., Monday, Tuesday, Wednesday, Thursday, Friday, Saturday, and Sunday), *mood* (i.e., bored, stressed, anxious/tense, irritable, frustrated, angry, sad, fatigued, disgusted, embarrassed, distressed, relaxed, proud, happy, or euphoric), and *type of alcoholic beverage* (i.e., beer, red wine, white wine, Sangria, Lambrusco, Spanish sparkling wine, cider, vermouth, cognac/brandy/sherry, herbal marc, anise, liquor, shots, carajillo [coffee with brandy], tequila, vodka, rum, gin, whisky, gin-tonic, mojito cocktail, or a cuba libre [rum-cola cocktail]). These variables were selected based on a literature review, as outlined in the introduction. Nevertheless, open items were included at the end of each subsection to allow other variables or specific cues to be added if considered relevant by participants. For each item, participants were asked to imagine a scene and report their desire to consume alcohol on a Likert scale ranging from 0 (no desire) to 4 (very high desire). For those variables where participants scored at least 1, they were asked to write in a text box about any associated stimuli (e.g., objects, people, and sounds).

### Procedure

The cross-sectional data for the present study was obtained in single sessions, during which the participants were informed about the scope of study. Patients were invited to participate in the study during their regular visits with their appointed psychiatrist or clinical psychologist at the Addictive Behaviors Unit. The craving questionnaire and the AUDIT (*M* = 15.19, *SD* = 9.73) were administered in random order.

### Data Analyses

To address the main objective of this exploratory study, various descriptive and frequency analyses were conducted to explore craving levels among patients with regards to the following variables: presence of others, situations, time of the day, day of the week, mood, and type of alcoholic beverage. To assess gender differences, independent student *t*-tests were conducted to compare alcohol craving experienced by men and women for all variables. Similarly, student *t*-tests were used to explore the relationship between patient age (≤45 years and ≥46 years) and alcohol craving reported on all variables measured with the self-administered craving questionnaire. All statistical analyses were carried out using IBM SPSS version 24 (IBM Corp., Armonk, NY, United States).

## Results

### Presence of Others

In terms of drinking habits and alcohol craving, presence of peers or drinking alone were highly significant. Patients reported that their greatest levels of craving were experienced when drinking alone (*M* = 1.31, *SD* = 1.26), followed by drinking with two or more friends (*M* = 1.31, *SD* = 1.10). Also, drinking with a friend was associated with increased alcohol craving (*M* = 1.17, *SD* = 1.032). By contrast, drinking with relatives (*M* = 0.72, *SD* = 1.02), drinking with a partner (*M* = 0.68, *SD* = 1.67), and drinking with co-workers (*M* = 0.57, *SD* = 0.96) seemed to trigger less alcohol craving.

### Situations

This factor highlighted the situations or contexts associated with higher alcohol craving in patients with AUD. Parties (*M* = 1.81, *SD* = 1.36) appeared to induce highest levels of alcohol craving, followed by restaurants (*M* = 1.63, *SD* = 1.19), bars (*M* = 1.55, *SD* = 1.38), pubs (*M* = 1.2, *SD* = 1.56), being at home (*M* = 1.15, *SD* = 1.08), and being in a club (*M* = 1.13, *SD* = 1.5). Lower levels of craving were experienced during meals (*M* = 1.01, *SD* = 1.07), being in the living room (*M* = 0.95, *SD* = 1.16,), or kitchen (*M* = 0.83, *SD* = 1.22), being in the work place (*M* = 0.55, *SD* = 1.10), being in the bedroom (*M* = 0.41, *SD* = 1), being in a supermarket (*M* = 0.36, *SD* = 0.81), or being in parks (*M* = 0.29, *SD* = 0.67).

### Time of the Day and Day of the Week

Drinking at night (*M* = 1.64, *SD* = 1.45) and during the afternoon (*M* = 1.32, *SD* = 1.31) were associated with increased alcohol craving compared to drinking at midday (*M* = 1.08, *SD* = 1.1) and in the morning (*M* = 0.75, *SD* = 1.14). Furthermore, patients experienced higher levels of alcohol craving as the week progressed, with levels peaking on a Saturday (*M* = 1.71, *SD* = 1.42), Friday (*M* = 1.63, *SD* = 1.38), and Sunday (*M* = 1.45, *SD* = 1.37). Participants also reported notably elevated cravings for alcohol on a Thursday (*M* = 1.23, *SD* = 1.25) and Wednesday (*M* = 1.15, *SD* = 1.21), compared to slightly lower craving levels reported on a Monday (*M* = 1.12, *SD* = 1.29) and Tuesday (*M* = 1.09, *SD* = 1.21).

### Mood

Depending on their mood, individuals experienced varying levels of alcohol craving. Patients reported that negative emotions such as being anxious or tense (*M* = 1.44, *SD* = 1.45), sad (*M* = 1.43, *SD* = 1.37), stressed (*M* = 1.37, *SD* = 1.55), frustrated (*M* = 1.36, *SD* = 1.56), irritable (*M* = 1.23, *SD* = 1.47), angry (*M* = 1.2, *SD* = 1.34), distressed (*M* = 1.2, *SD* = 1.44), or disgusted (*M* = 1.09, *SD* = 1.37) were associated with alcohol cravings. However, positive emotions such as feeling euphoric (*M* = 1.21, *SD* = 1.34) and happy (*M* = 1.09, *SD* = 1.19) were also associated with craving for alcohol. Lower craving self-reports were associated with boredom (*M* = 0.92, *SD* = 1.42), relaxation (*M* = 0.71, *SD* = 1.11), fatigue (*M* = 0.67, *SD* = 1.27), pride (*M* = 0.59, *SD* = 1.14), or embarrassment (*M* = 0.44, *SD* = 1.08).

### Type of Alcoholic Beverage

The following alcoholic drinks were most associated with cravings: beer (*M* = 1.87, *SD* = 1.41), red wine (*M* = 1.15, *SD* = 1.32), and whisky (*M* = 0.87, *SD* = 1.3). Lower craving levels were associated with gin-tonic (*M* = 0.85, *SD* = 1.13), Spanish sparkling wine (*M* = 0.79, *SD* = 1.2), white wine (*M* = 0.69, *SD* = 1.07), vermouth (*M* = 0.64, *SD* = 1.09), shots (*M* = 0.63, *SD* = 1.07), gin (*M* = 0.6, *SD* = 1.18), mojitos (*M* = 0.52, *SD* = 1.08), herbal marc (*M* = 0.47, *SD* = 1.03), rum (*M* = 0.45, *SD* = 1.0), Cuba-libre (*M* = 0.45, *SD* = 1), carajillo (*M* = 0.44, *SD* = 0.94), cognac/brandy/sherry (*M* = 0.43, *SD* = 0.91), tequila (*M* = 0.43, *SD* = 1.02), vodka (*M* = 0.41, *SD* = 0.98), Sangria (*M* = 0.36, *SD* = 0.84), anise (*M* = 0.36, *SD* = 0.89), liquor (*M* = 0.31, *SD* = 0.87), Lambrusco (*M* = 0.28, *SD* = 0.81), and cider (*M* = 0.24, *SD* = 0.72).

#### Specific Cues

The patients reported specific cues on the open items included at the end of each subsection of the questionnaire. Drinking with peers appeared as a triggering factor in patients with AUD. In terms of drinking with a friend, 21.3% of the patients reported that this elicited alcohol craving. Similarly, regarding drinking with two or more friends, 22.7% of patients reported that this triggered alcohol craving. In the living room setting, 13.3% of patients reported that the presence of alcoholic beverages increased craving, and 18.7% of patients reported that the presence of alcoholic beverages in the house elicited cravings. Regarding restaurant settings, 14.7% stated that the presence of food increased alcohol cravings due to the association between food and alcohol intake.

#### Gender and Age

In terms of the presence of others, there were statistically significant differences between men and women regarding drinking alone [*t*_(72)_ = 2.642, *p* < 0.05], with women tending to experience greater alcohol craving (*M* = 1.87, *SD* = 1.21) compared to men (*M* = 1.06, *SD* = 1.22). Similarly, there was a significant difference between men and women regarding drinking with partner [*t*_(72)_ = 2.274, *p* < 0.05]. Women also tended to experience higher levels of craving when drinking with their partner (*M* = 1.09, *SD* = 1.37) compared to men (*M* = 0.49, *SD* = 0.85). There were no statistically significant differences between males and females (*p* > 0.05) regarding variables such as drinking with a friend, with two or more friends, with relatives, or with co-workers.

Regarding situations, there was a statistically significant difference between men and women with regards to drinking in restaurants [*t*_(72)_ = 2.073, *p* < 0.05], with women experiencing greater craving levels (*M* = 2.04, *SD* = 1.33) compared to men (*M* = 1.43, *SD* = 1.1). Similar patterns were found regarding drinking in the kitchen [*t*_(72)_ = 2.483, *p* < 0.05, *M*_women_ = 1.35, *SD* = 1.26 vs. *M*_men_ = 0.61, *SD* = 1.15], in the work place [*t*_(72)_ = 2.645, *p* < 0.05, *M*_women_ = 1.04, *SD* = 1.46 vs. *M*_men_ = 0.33, *SD* = 0.84], in the bedroom [*t*_(72)_ = 2.984, *p* < 0.005, *M*_women_ = 1.35, *SD* = 1.26 vs. *M*_men_ = 0.61, *SD* = 1.15], and at the supermarket [*t*_(72)_ = 1.999, *p* < 0.05, *M*_women_ = 0.61, *SD* = 1.11 vs. *M*_men_ = 0.22, *SD* = 0.57]. However, there were no statistically significant gender differences regarding drinking in the living room, bar, pub, park, club, parties, at home or during meals (*p* > 0.05).

Women experienced significantly greater craving levels during morning time (*M* = 1.17. *SD* = 1.30) compared to men (*M* = 0.57, *SD* = 1.02) [*t*_(72)_ = 2.158, *p* < 0.05]. Similarly, women generally experienced significantly greater alcohol craving at midday (*M* = 1.52, *SD* = 0.94) compared to men (*M* = 0.88, *SD* = 1.12) [*t*_(72)_ = 2.370, *p* < 0.05]. No statistically significant differences were found concerning drinking during the afternoon or at night (*p* > 0.05), or drinking throughout the week (*p* > 0.05).

In terms of mood, there were significant differences regarding feelings of anxiety or tension [*t*_(72)_ = 2.893, *p* = 0.005], sadness [*t*_(72)_ = 3.249, *p* < 0.005], stress [*t*_(72)_ = 2.241, *p* < 0.05], frustration [*t*_(72)_ = 2.723, *p* < 0.05], distress [*t*_(72)_ = 3.467, *p* = 0.001], disgust [*t*_(72)_ = 2.549, *p* < 0.005], fatigue [*t*_(72)_ = 2.75, *p* < 0.005], and embarrassment [*t*_(72)_ = 2.576, *p* < 0.005]. Compared with men, women experienced greater cravings when feeling anxious or tense (*M*_women_ = 2.13, *SD* = 1.48 vs. *M*_men_ = 1.12, *SD* = 1.35), sad (*M*_women_ = 2.17, *SD* = 1.26 vs. *M*_men_ = 1.12, *SD* = 1.30), stressed (*M*_women_ = 1.96, *SD* = 1.63 vs. *M*_men_ = 1.10, *SD* = 1.47), frustrated (*M*_women_ = 2.09, *SD* = 1.59 vs. *M*_men_ = 1.06, *SD* = 1.46), distressed (*M*_women_ = 2, *SD* = 1.53 vs. *M*_men_ = 0.82, *SD* = 1.26), disgusted (*M*_women_ = 1.7, *SD* = 1.42 vs. *M*_men_ = 0.84, *SD* = 1.28), fatigued (*M*_women_ = 1.26, *SD* = 1.6 vs. *M*_men_ = 0.41, *SD* = 1.02) and embarrassed (*M*_women_ = 0.91, *SD* = 1.53 vs. *M*_men_ = 0.24, *SD* = 0.73). No statistically significant differences were found regarding emotional states like boredom, irritability, anger, relaxation, pride, happiness or euphoria (*p* > 0.05).

There were significant differences in craving levels between men and women regarding the type of alcoholic beverages, including white wine [*t*_(72)_ = 3.362, *p* = 0.001; *M*_women_ = 1.26, *SD* = 1.28 vs. *M*_men_ = 0.41, *SD* = 0.85], vermouth [*t*_(72)_ = 3.155, *p* = 0.005; *M*_women_ = 1.22, *SD* = 1.38 vs. *M*_men_ = 0.39, *SD* = 0.85], and Spanish sparkling wine [*t*_(72)_ = 3.055, *p* < 0.005; *M*_women_ = 1.35, *SD* = 1.36 vs. *M*_men_ = 0.49, *SD* = 0.98]. Although lower ratings of craving were reported regarding alcoholic beverages like Mojito, vodka, Sangria or cider, women were more likely to prefer these drinks than men [Mojito, *t*_(72)_ = 2.511, *p* < 0.05; *M*_women_ = 0.96, *SD* = 1.26 vs. *M*_men_ = 0.33, *SD* = 0.84; vodka, *t*_(72)_ = 2.448, *p* < 0.05; *M*_women_ = 0.83, *SD* = 1.3 vs. *M*_men_ = 0.33, *SD* = 0.9; Sangria, *t*_(72)_ = 2.305, *p* < 0.05; *M*_women_ = 0.70, *SD* = 1.18 vs. *M*_men_ = 0.22 *SD* = 0.61; and cider, *t*_(72)_ = 2.243, *p* < 0.05; *M*_women_ = 0.52, *SD* = 1.08 vs. *M*_men_ = 0.12, *SD* = 0.47]. No statistically significant differences were found in other craving self-reports of alcoholic beverages. A comprehensive overview of the results is shown in Table 1.

**Table 1:** Craving levels as a function of gender.

	Mean craving level (*SD*)		
	Gender		
Significant variables	Males	Females	*t*
Drinking alone	1.06 (1.22)	1.87 (1.21)	2.64*
Drinking with two or more friends	1.2 (1.07)	1.48 (1.12)	1.02
Drinking with a friend	1.1 (0.96)	1.3 (1.18)	0.79
Drinking at a party	1.76 (1.35)	1.87 (1.42)	0.3
Drinking in a restaurant	1.43 (1.1)	2.04 (1.33)	2.07ˆ*
Drinking in a bar	1.41 (1.34)	1.78 (1.47)	1.06
Drinking in a pub	1.04 (1.45)	1.48 (1.75)	1.12
Drinking at home	1 (0.98)	1.43 (1.27)	1.6
Drinking at night	1.49 (1.43)	1.96 (1.52)	1.27
Drinking during the afternoon	1.29 (1.27)	1.39 (1.46)	0.29
Drinking on a Friday	1.51 (1.33)	1.83 (1.49)	0.9
Drinking on a Saturday	1.67 (1.4)	1.78 (1.47)	0.32
Drinking while anxious/tense	1.12 (1.35)	2.13 (1.48)	2.89ˆ*
Drinking while sad	1.12 (1.3)	2.17 (1.26)	3.24ˆ*
Drinking while stressed	1.1 (1.47)	1.96 (1.63)	2.24*
Drinking while frustrated	1.06 (1.46)	2.09 (1.59)	2.72*
Drinking beer	1.96 (1.31)	1.65 (1.64)	0.8
Drinking red wine	1.06 (1.27)	1.3 (1.46)	0.73
Drinking whisky	0.98 (1.31)	0.65 (1.3)	0.99


*^∗^p < 0.05.*

In terms of age, the only statistically significant differences between patients aged ≤45 years and patients aged ≥46 years were found regarding craving levels in pubs [*t*_(70)_ = 1.108, *p* = 0.001; *M*_<45_ = 2.29, *SD* = 1.53 vs. *M*_>46_ = 0.85, *SD* = 1.43] and clubs [*t*_(70)_ = 2.244, *p* < 0.05, *M*_<45_ = 1.82, *SD* = 1.62 vs. *M*_>46_ = 0.91, *SD* = 1.41]. Patients aged ≤45 years also experienced greater cravings at night (*M*_<45_ = 2.29, *SD* = 1.40) compared with patients aged ≥46 years [*t*_(70)_ = 2.055, *p* < 0.05, *M*_>46_ = 1.47, *SD* = 1.45]. Finally, patients aged ≤45 years displayed a preference for Sangria drink [*t*_(70)_ = 2.638, *p* < 0.05; *M*_<45_ = 0.82, *SD* = 1.07 vs. *M*_>46_ = 0.22, *SD* = 0.73], and liquor [*t*_(70)_ = 2.108, *p* < 0.05; *M*_<45_ = 0.71, *SD* = 1.31 vs. *M*_>46_ = 0.20, *SD* = 0.67].

## Discussion

The main study objective was to identify triggers of alcohol craving in a clinical sample of patients with diagnosed AUD. We were particularly interested in determining which specific cues and contexts were most commonly reported in patients with AUD. The results of this study will now be used to create VR environments to implement VR-CET for the treatment of patients with AUD.

This study indicated that some contexts and stimuli (e.g., mood, time of day, day of the week, and presence of others) were associated with greater self-reported levels of craving. The contexts associated with the greatest alcohol cravings were being at a party, in a restaurant, in a bar, in a pub, and in a house. This is consistent with previous studies (Lee et al., 2007; Nees et al., 2012; Qureshi et al., 2017), suggesting that these truly reflect the most common situations associated with craving and alcohol consumption. Of note, the presence of food has been reported to increase alcohol craving, particularly because of the association between food intake and alcohol consumption. For example, Lee et al. (2009) implemented VR scenarios like pubs and restaurant in their protocol to reduce alcohol cravings. Similarly, VR bars have been used to explore alcohol craving (Ghiţă et al., 2017) or to decrease levels of alcohol cravings in VR-CET (Lee et al., 2007). Generally, these environments involve a wide range of stimuli (e.g., alcohol bottles and glasses), social interactions, and perceived leisure time. In our study, the urge to consume alcohol was also associated with meals and being in the living room, but self-reported levels of craving were lower. The most common craving-related beverages reported by the AUD group were beer, wine, and whisky, and their presence across different environments (e.g., living room or in the house) triggered alcohol cravings. These data will be important to creating useable VR environments for CET. The goal of VR-CET is to expose patients to relevant scenarios gradually, seeking to extinguish conditioned responses. This will be facilitated by allowing patients to explore different situations that elicit different levels of alcohol craving in naturalistic environments.

Our data also suggested that the presence of other individuals play a key role in eliciting alcohol craving. Interestingly, our results showed that drinking alone and drinking with two or more friends were equally associated with greater craving levels. Consistent with research by Ryan et al. (2010), patients diagnosed with AUD experience alcohol craving in both situations. The time of day was also important trigger of craving. Notably, drinking at night was associated with the greatest levels of alcohol craving, but our data showed that patients with AUD generally experienced urges to consume alcohol regardless of the time of the day. Late night drinking, however, has been associated with drinking in public spaces and has emerged as a high risk trigger for heavy drinking episodes (Kuntsche and Cooper, 2010). Concerning the day of the week, drinking during weekends (Friday, Saturday, or Sunday) was associated with greater craving levels compared with other weekdays. This supports previous research (Beets et al., 2009; Kuntsche and Cooper, 2010), which indicated that individuals tended to consume higher amounts of alcohol during weekends, particularly due to its relationship to leisure time, a lack of work commitments, and parties.

Mood has frequently been associated with alcohol consumption because of the important role that emotions play in alcohol intake, dependence, and abstinence (Heinz et al., 2003; Birch et al., 2004; Sinha, 2008; Witkiewitz et al., 2011). Both positive and negative emotional states are known to be heavily involved in craving for the rewarding effects of alcohol intake, so this implicit motivational learning is strongly associated with alcohol-related stimuli (Heinz et al., 2003). Interestingly, our data indicated that AUD patients generally self-reported greater cravings with negative emotions like anxiety or tension, sadness, stress, frustration or irritability. This is consistent with previous research showing that negative emotional states tend to predict alcohol consumption (Townshend and Duka, 2005) and precipitate relapse after treatment for AUD (Sinha et al., 2009).

### Clinical Application

This study was performed as a part of a larger project aiming to develop and validate the use of VR-based CET in patients with AUD who are resistant to classical treatment and experience several relapses despite treatment. We were particularly interested in the interplay between different self-reported ratings of craving related to preferred alcoholic drinks and contexts. However, this also served as an opportunity for further, preliminary, research. Therefore, based on the results of this study, we created the “ALCO-VR” software.

In the ALCO-VR software, we concentrated on the first four physical environments reported to elicit greater craving levels among patients (restaurant, bar, pub, and house). Because the party variable could be either a physical setting or a personal valence in a celebration, we simulated a party within the VR pub environment by adding a party background. Considering the other variables, we created different VR environments, taking care to include social interactions, different alcoholic beverages, or different times of day. For example, the house VR environment at night, with no social interaction would be expected to be most stimulating because drinking alone was highly correlated with alcohol craving. A restaurant VR setting was designed during daylight and with social interaction; for this, a naturalistic environment was simulated in which avatars were moving, eating, and drinking, with food-related stimuli on tables. For the VR bar setting, we adopted a generic concept of a bar. This was set during daylight with social interaction and consisted of a bar with a wide variety of alcoholic beverages in the background. Participants could sit at the bar on a stool or at a table with other drinking avatars. Finally, the pub VR setting was set at night and simulated a party with lots of drinking avatars, many alcoholic beverages, and bottles placed within the environment.

**FIGURE 1:**
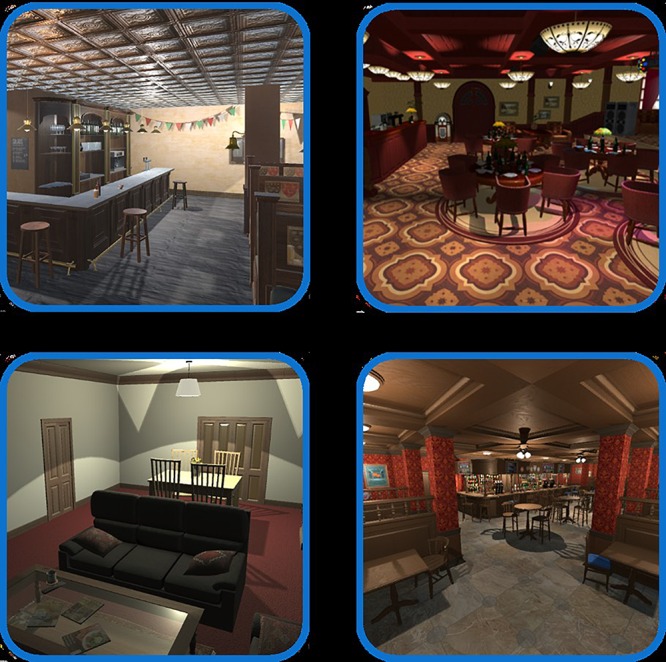
“ALCO-VR.” Pictures of the VR environments.

As reported by the participants in our study, drinking with two or more friends elicited alcohol cravings, so the restaurant, bar, and pub settings all included drinking avatars. Therefore, we created four VR environments in total: two were set during daylight (restaurant and bar) and two were set at night (house and pub); and one included no avatars (house) while the other three included many avatars (restaurant, bar, and pub). All these environments included different alcohol-related stimuli, such as bottles and glasses containing alcohol. Screenshots of the VR environments are shown in Figure 1.

The ALCO-VR software will be implemented in a VR-CET protocol to treat patients diagnosed with AUD, with the hope that the advantages of the VR technology will facilitate the treatment of mental health problems. In VR, such naturalistic environments can be recreated based on patient experience. VR exposure to relevant cues and contexts also provides a safe and secure, yet a flexible approach for *in vivo* exposure. VR-CET cannot only help patients to improve their coping skills in future situations but also allow clinicians to control the variables and inputs in an environment. This will also allow exploration of the interplay between preferred alcoholic drinks and alcohol-related contexts based on different ratings for craving levels elicited by each drink and context.

## Conclusion

This study was part of a larger project aiming to develop and validate CET based on VR technology for patients with AUD who are resistant to classical treatment. The identified triggers were used to develop relevant VR environments for CET, and further research is ongoing to implement our findings. However, this study had several limitations that warrant consideration. First, only a few participants completed the open items in the questionnaire, and most did not specify which cues they considered important in a situation or context. Other limitations were that self-reported craving was assessed by questionnaire and that self-reported cravings tended to be low in AUD patients. This may be explained by the fact that patients with AUD have a tendency to avoid or to minimize their condition (Forsyth et al., 2003), and should not be discounted. Our sample included fewer female than male participants and future studies should adjust this issue. Environments used in VR-CET may help to counteract issues of avoidance, may help to elicit different levels of alcohol craving, and may facilitate the generalization of therapeutic effects to real-life settings.

## Author Contributions

All authors have made a substantial contribution to the work and have approved the manuscript for publication.

## Conflict of Interest Statement

The authors declare that the research was conducted in the absence of any commercial or financial relationships that could be construed as a potential conflict of interest.
